# First-episode psychosis and migration in Italy (PEP-Ita migration): a study in the Italian mental health services

**DOI:** 10.1186/1471-244X-14-186

**Published:** 2014-06-23

**Authors:** Ilaria Tarricone, Mauro Braca, Fabio Allegri, Giuseppe Barrasso, Antonello Bellomo, Vanna Berlincioni, Bernardo Carpiniello, Alessio Ceregato, Marco Conforti Donati, Samuele Defilippi, Valeria Del Vecchio, Corrado De Rosa, Luigi Ferrannini, Silvia Ferrari, Maria Antonietta Furio, Carla Gramaglia, Caterina La Cascia, Mario Luciano, Alice Mulè, Marcello Nardini, Francesca Podavini, Diego Primavera, Corinna Reggianini, Marco Rigatelli, Orlando Todarello, Elena Turella, Antonio Ventriglio, Patrizia Zeppegno, Andrea Fiorillo, Domenico Berardi

**Affiliations:** 1 Department of Medical and Surgical Sciences, Bologna University, Bologna, Italy; 2 Department of Mental Health and Pathological Addictions, Local Health Trust, Bologna, Italy; 3 Andria Community Mental Health Centre, Andria, BAT, Italy; 4 Department of Clinical and Experimental Sciences, Section of Psychiatry and Clinical Psychology, University of Foggia, Foggia, Italy; 5 Department of Brain and Behavioral Sciences, University of Pavia, Pavia, Italy; 6 Clinica Psichiatrica - Università degli Studi di Cagliari, Cagliari, Italy; 7 Mental Health Department A.S.L. TO4, Chivasso, TO, Italy; 8 Department of Psychiatry, University of Naples SUN, Napoli, Italy; 9 Department of Mental Health ASL 3 Liguria, Genova, Italy; 10 U.O. Psichiatria Ospedaliero-Universitaria Modena Centro, University of Modena and Reggio Emilia, Modena, Italy; 11 U.O. di Psichiatria - Azienda Ospedaliero Universitaria “Consorziale Policlinico” Bari, Bari, Italy; 12 Dipartimento di Scienze Mediche di Base, Neuroscienze ed Organi di Senso, Università degli Studi Aldo Moro di Bari, Bari, Italy; 13 SC Psichiatria, AOU Ospedale Maggiore della Carità, Dipartimento di Medicina Traslazionale, Università del Piemonte Orientale Amedeo Avogadro, Novara, Italy; 14 Psychiatric Unit, Azienda Ospedaliera Universitaria Policlinico “P. Giaccone” Palermo, Palermo, Italy; 15 Department of Experimental Biomedicine and Clinical Neuroscience, Section of Psychiatry, University of Palermo, Palermo, Italy; 16 U.O.A. di Psichiatria di Camposampiero (PD), Camposampiero, PD, Italy

**Keywords:** Migrants, First-episode psychosis, Italy, Risk factors, Protective factors

## Abstract

**Background:**

It has been frequently reported a higher incidence of psychotic disorders in immigrants than in native populations. There is, however, a lack of knowledge about risk factors which may explain this phenomenon. A better understanding of the causes of psychosis among first-generation migrants is highly needed, particularly in Italy, a country with a recent massive migration.

**Methods/Design:**

The “Italian study on first-episode psychosis and migration (PEP-Ita)” is a prospective observational study over a two-year period (1 January 2012–31 December 2013) which will be carried out in 11 Italian mental health centres. All participating centres will collect data about all new cases of migrants with first-episode psychosis. The general purpose (“core”) of the PEP-Ita study is to explore the socio-demographic and clinical characteristics, and the pathways to care of a population of first-episode psychosis migrants in Italy. Secondary aims of the study will be: 1) to understand risk and protective factors for the development of psychotic disorders in migrants; 2) to evaluate the correlations between psychopathology of psychotic disorders in migrants and socio-demographic characteristics, migration history, life experiences; 3) to evaluate the clinical and social outcomes of first-episode psychoses in migrants.

**Discussion:**

The results of the PEP-Ita study will allow a better understanding of risk factors for psychosis in first-generation migrants in Italy. Moreover, our results will contribute to the development of prevention programmes for psychosis and to the improvement of early intervention treatments for the migrant population in Italy.

## Background

Several studies report a higher incidence of psychotic disorders in immigrant populations when compared to native populations [1-4]. Epidemiological studies about the incidence of severe mental disorders in migrants, as well as the identification of specific vulnerabilities and stressors associated with migration, have significantly contributed to the development of models explaining the etiopathogenesis of psychosis in this population. According to the sociodevelopmental model of Morgan et al. [3], the exposure to negative life events can impact on brain development (in particular on the dopaminergic system), as well as on personal sensitivity to stress, inducing a state of persistent vulnerability to psychosis (consisting of social bias, psychotic-like experiences and affective disorders). These factors are thought to weigh on from the perinatal period and through different life stages [5,6]. During childhood, incidents such as head injuries, infectious processes, separation from parents, lack of social relationships, psychological, physical or sexual abuse and bullying are likely to be associated with the development of psychosis [7-9]. During adolescence, cannabis abuse is considered an ‘at risk’ behavior, along with other subjective experiences, such as discrimination and bullying at school, which appear to increase the individual risk of developing psychotic disorders [10,11]. In adulthood, relevant factors that seem to be associated with the onset of psychosis are social exclusion, racial discrimination, discrepancy between expectations and achievements, lack of confidants/emotional relationships, and loneliness [12,13].

Risk factors for the development of psychosis are not only at an individual level, but also in the social area [14]. It has been shown that the risk of developing psychosis is higher in urban areas [15-17], but the meaning of this association remains unclear; it is assumed that one reason could be social isolation which occurs in large urban areas [18,19]. In addition, other factors, such as exposure to pollutants, lifestyle, capacity/ability to relate to environment, and restricted working environments, may represent other risk factors. Indeed, the quality and the resources of the social structure in which a person lives (the so-called “social capital”) may also significantly affect their risk of developing psychosis [20]. Some studies have shed light on how urban areas which are characterized by the presence of strong socio-economic disparities show a higher incidence of psychosis [21].

The migratory process is a life experience that puts the individual at an increased risk of dealing with the events and factors listed above [22]. In fact, the events experienced by migrants with psychotic onset may be considered a negative life event itself. A better understanding of this process may constitute the key to explain the higher risk of psychosis in these patients.

The high incidence of psychosis in migrants has been described by Morgan [23] as ‘a tragedy of public health’. Moreover, it has been reported that access to mental health care for migrants can be particularly difficult, notwithstanding the high psychiatric morbidity that exists in this population [24-26].

Only few studies have investigated the risk factors for the onset of psychotic disorders in migrants in Italy [4,27]. Here health care is provided to the population by the Italian National Health Service, which is built like the British National Health Service. All the population has unlimited health care coverage, which is provided by “Local Health Units”, each responsible for a geographically defined catchment area. Access to health services is generally free of charge. Accordingly with the 1978 reform, psychiatric care is delivered by general hospital psychiatric wards for acute admissions, and community mental health centres (CMHC) providing psychiatric care to geographically defined areas. Non-resident people such as temporary migrants can access to care as much as resident population for urgent and/or necessary cases. CMHCs (an average of 1.81 services per 150,000 population) deliver individual consultations and domiciliary care activities, keep contacts with other health and social agencies and provide emergency interventions. They have a multidisciplinary staff, including psychiatrists, psychologists, social workers, nurses, and educators. Migrants are cared by the same CMHCs of the general population, and cultural-competent activities are provided on demand (e.g. interpreters, cultural mediators, social/legal support, etc.) [28,29]. So structured, such an organization would facilitate access to care for migrants presenting first-episode psychosis in Italy; yet, previous studies demonstrated that migrants with mental disorders might follow different and more complex pathways to care [26].

Migration to Italy is a relatively recent phenomenon, mainly developed in the last 20 years and still increasing (+8.2% in 2013) [30]. At the beginning of 2013, migrants represented 7.4% of the resident population. Migrants come to Italy from any continent, more frequently from European countries (both EU, 27.4% and non-EU, 23.4%); 22.1% from Africa, followed by Asian (18.8%) and American (8.3%) migrants. The most represented countries of origin are Romania (21.2%), Albania (10.6%), Morocco (9.9%), China (4.6%), Ukraine (4.4%), Philippines (2.9%), Moldova (2.9%), India (2.6%), Poland (2.4%) and Tunisia (2.3%) [31]. Most migrants in Italy live in the Northern regions (61.8%) and emigrated to find a job, though the reason for migration has suddenly changed in the recent years: in 2012, family reasons (81.322 migrants) became more frequent than work reasons (52.328 migrants), probably because of the progressive decrease of job offer in Italy and the stagnation in labour market [30]. Also, migrants who come to Italy are frequently asylum-seekers: in 2012 about 60.000 people have reached our coasts from Tunisia and Lybia.

Based on these premises, this research project was promoted in order to understand risk factors for psychosis in migrants, which could be targeted by specific psychosocial interventions.

The study will be carried out by 11 study centres across Italy and will be coordinated by the Bologna Transcultural Psychiatric Team (BoTPT) [27,32-34]. The BoTPT is a team of the University of Bologna, which works closely with the Bologna Department of Mental Health and Pathological Addictions in its provision of cultural-competent treatment care of mental disorders. It also performs consultation and liaison interventions for other urban services that deal with social and health problems of migrants and their families, such as local social services and voluntary organizations. The BoTPT also collaborates with other psychiatric services and University departments in Italy on the topic of migration and mental health. These collaborations are performed in order to understand the occurrence of different disorders in relation to specific cultural contexts, the different migratory movements in different areas, as well as the specific models of care. An initial investigation involving four Italian centres revealed important differences in pathways to care and the socio-demographic characteristics of migrants attending psychiatric services [26].

More recently, Bologna has become part of the EU-GEI European project (European Community’s Seventh Framework Program, grant agreement No. HEALTH-F2-2009-241909, Project EU-GEI: European Network of National Schizophrenia Networks Studying Gene-Environment Interaction) coordinated by the University of Maastricht. Bologna’s participation in EU-GEI and the Work Package 2 (coordinated by Craig Morgan and Robin Murray from the Institute of Psychiatry – King’s College, London) allowed the use of EU-GEI’s methods both in Bologna and in the independent network of Italian mental health centres which are taking part to the Italian study on first-episode psychosis and migration (PEP-Ita migration study).

The Italian study on first-episode psychosis and migration (PEP-Ita) brings together spontaneous and independent research experiences in various Italian mental health centres (psychiatric wards, psychiatric consultation-liaison services and/or CMHCs) with the purpose to study the incidence of psychosis in migrants in Italy. The general aim (“core”) of the study is to explore the socio-demographic and clinical characteristics, and the pathways to care of a population of first-episode psychosis (FEP) migrants in Italy. The study has the following secondary aims: 1) to understand risk and protective factors for the development of psychotic disorders in migrants; 2) to evaluate the link between psychopathology of psychotic disorders in migrants and socio-demographic characteristics, migration history, life experiences; 3) to assess the clinical and social outcome of FEP in migrants.

## Methods

### Study design

The PEP-Ita migration project is a prospective observational study over a two-year period (1 January 2012–31 December 2013). All mental health centres participating in the study (Table 1) will collect data on all new cases of migrants with first-episode psychosis and, if their catchment areas will allow it, they will evaluate the incidence of psychosis in migrants. Data collection will be carried out through a central computerized system.

**Table 1:** Italian study on first-episode psychosis and migration (PEP-Ita migration) - Mental Health Centres network

**Study centers**	** Mental Health Services and Universities**
***Coordinating centres***	
**Bologna**	• Bologna Transcultural Psychiatric Team (BoTPT) - Department of Medical and Surgical Sciences, University of Bologna
	• Department of Mental Health and Pathological Addictions, Local Health Trust, Bologna
**Napoli**	• Department of Psychiatry, University of Naples SUN
***Participant centres***	
Andria*	• Andria Community Mental Health Centre
Bari*	• U.O. di Psichiatria - Azienda Ospedaliero Universitaria “Consorziale Policlinico” Bari
	• Dipartimento di Scienze Mediche di Base, Neuroscienze ed Organi di Senso, Università degli Studi Aldo Moro di Bari
**Bologna**	• Department of Medical and Surgical Sciences, University of Bologna
	• Department of Mental Health, Ausl Bologna
Cagliari*	• Clinica Psichiatrica - Università degli Studi di Cagliari
**Camposampiero (PD)**	• U.O.A. di Psichiatria di Camposampiero (PD)
**Chivasso (TO)**	• Department of Mental Health A.S.L. TO4
**Foggia**	• Department of Clinical and Experimental Sciences, Section of Psychiatry and Clinical Psychology, University of Foggia
**Modena**	• U.O. Psichiatria Ospedaliero-Universitaria Modena Centro, University of Modena and Reggio Emilia
**Novara**	SC Psichiatria, AOU Ospedale Maggiore della Carità, Dipartimento di Medicina Traslazionale, Università del Piemonte Orientale Amedeo Avogadro, Novara, Italy
**Palermo**	• Psychiatric Unit, Azienda Ospedaliera Universitaria Policlinico “P. Giaccone” Palermo
	• Department of Experimental Biomedicine and Clinical Neuroscience, Section of Psychiatry, University of Palermo
**Pavia**	• Department of Brain and Behavioral Sciences, University of Pavia

*Research protocol currently under approval.

**Figure 1:**
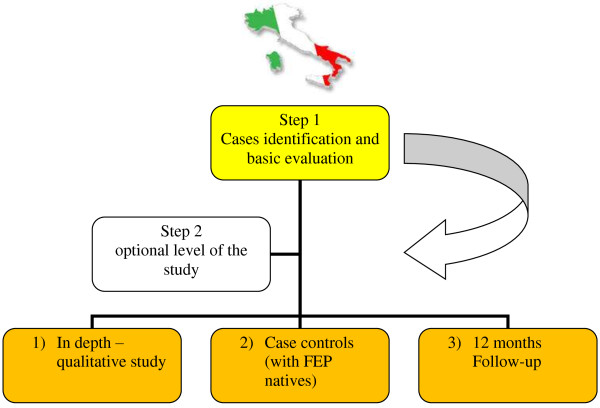
Study design.

Based on data from the Italian National Institute of Statistics (ISTAT) [35], with a whole catchments area of 2.135.145 inhabitants and 6.5% of migrants, and considering a conservative estimate of yearly incidence cases of 20/100.000 among Italians and 40/100.000 among migrants, as already reported by previous estimates [4], we will expect to recruit 111 FEP migrants cases by year 2. Twenty-four months after the survey period, we will conduct a leakage study to identify any subject that may have been missed during the critical data collection period. In order to do so, we will review all new mental health service registration forms and will interrogate the computerized information systems.Besides the main study design, optional levels (Figure 1) of the research are: 1) an in-depth assessment of FEP migrant patients through the administration of a battery of research instruments (listed below); 2) a case–control study, the control group consisting in FEP native patients consecutively attending psychiatric services in the same catchment areas and in the same study period as FEP migrant patients; 3) a follow-up study, which will perform a three- and a twelve-months follow-up assessment.

### Study population

The study will include untreated first-generation migrants with FEP who seek treatment for the first time at participating mental health centres during the recruitment period. As the World Health Organization, we define “migrants” people who move from one area to another for varying periods of time and reasons [36]; specifically, with “first-generation migrants” we mean people who were born in a foreign country and have moved to Italy for any period or reason: thus we refer to international migrants and not to internal ones. In order to ensure that patients have not yet presented and been treated for a psychotic episode, an accurate medical history will be recorded together with patients and, where necessary, with their families. Previous use of medications or other therapeutic strategies will be the object of a strict survey.

The inclusion criteria for the study population are: 1) diagnosis of a psychotic episode, defined on the basis of the criteria currently in use (Diagnostic and Statistical Manual of Mental Disorders - DSM-IV-TR) [37]; 2) age between 18 and 64; 3) patients living in the study area of the involved mental health centres; 4) patients who accept to participate in the study and sign a valid informed consent. Patients will be excluded in case of comorbid mental retardation, moderate or severe, based on the criteria in current use (Diagnostic and Statistical Manual of Mental Disorders - DSM-IV-TR) [37]. Patients will be assessed by the WAIS-III short form [38]. The inclusion criteria for control native patients are the same as migrants: thus, all native patients consecutively presenting a first-episode psychosis and admitted to the designated psychiatric services for each study centre in the recruitment period (1 January 2012–31 December 2013) will be recruited as controls. Because of these recruitment criteria, control native patients will not be matched to migrant patients, and we will not be able to predict the number of controls until the recruitment process is concluded.

### Assessment

For each patient, the following information will be collected: 1. Informed consent; 2. Socio-demographic data (gender, birth date and place, ethnicity, marital status, educational level, employment status, housing status). As regards the optional levels of study, the evaluations will be performed by the use of a wide series of tests derived from the EU-GEI protocol [39] and by other research experiences of the participating centres.

1. *Nottingham Onset Schedule: Duration of Untreated Psychosis* version (NOS-DUP) [40] to examine the duration of untreated psychosis;

2. *Bologna Pathway to Care Scale* (BPCS) to provide information on routes of access to mental health care (general practitioners, patients’ direct access, family members’ involvement, other psychiatric services, etc.).

3. *Bologna Migration History Questionnaire and Social Integration Interview* (BO-MHQ) [4] for the study of the history of migration and the experiences related to it;

4. Association for Methodology and Documentation in Psychiatry (AMDP) [41] and Operational Criteria Checklist (OPCRIT) [42,43] for a thorough psychopathological rating;

5. *Premorbid Adjustment Scale* (PAS) [44] to evaluate the premorbid adjustment;

6. *Childhood Experiences of Care and Abuse Questionnaire* (CECA-Q) [45] and *Childhood Trauma Questionnaire* (CTQ) [46] to assess the presence of childhood trauma in the history of the patient;

7. *Amended Bullying Questionnaire*[47] to assess acts of bullying by peers (emotional, psychological or physical violence) inflicted on the subject before the age of 17;

8. *Harvard Trauma Questionnaire* (HTQ) [48] to investigate traumatic experiences that occurred during adult life;

9. *The Built Environment Assessment Tool* (BEAT) [49] allows the classification of the area of residence of the subject on the basis of a set of architectural evaluation criteria and available infrastructure.

10. *Discrimination Questionnaire*[50], a modified 12-item scale for significant and everyday elements of interpersonal discrimination;

11. *Cannabis Experiences Questionnaire* (CEQ) [51] to investigate the intake of cannabis and the subjective experience which results;

12. *Brief Core Schema Scales* (BCSS) [52] for the evaluation of positive and negative perceptions of self and others;

13. *Community Assessment of Psychic Experience* (CAPE) [53] to assess the schizotropical vulnerability;

14. *Devaluation of Consumers Families Scale* (DCFS) and *Devaluation of Consumers Scale* (DCS) [54] to measure the stigma perceived by service users and their families;

15. *Schedules for Clinical Assessment in Neuropsychiatry* (SCAN) [55] to obtain a standardized diagnosis and assess the psychopathology in detail;

16. *Global Assessment of Functioning Scale* (GAF) [35], Axis V of the DSM-IV, and *Schedule for the Deficit Syndrome* (SDS) [56] for the evaluation of the overall operation;

17. *Medical lists*: list of drugs taken at the time of evaluation.

Assessments will be carried out by psychiatrists or clinical psychologists appropriately trained in the use of the above-mentioned assessment instruments. The full assessment takes approximately 3 hours and it will be conducted within 2 to 4 sessions. In case of relevant linguistic barriers, a cultural mediator will join the clinical researcher: however, research questionnaires have been translated into several languages (English, French, German, Dutch, Spanish, Portuguese, Turkish, Serbian) through a back-translation process. This will help the direct assessment of the patient, confining the presence of cultural mediators only to those cases where migrants have significant linguistic barriers and instruments in their own language are not available. The level of agreement among professionals will be assessed after training on simulated cases through Cohen’s kappa coefficient.

### Online database

Data will be entered into a computerized database, which will be accessible online by all centres participating in the study. The computerized system will have the following characteristics: 1) usability: the proposed software is characterized by immediacy and effectiveness in data entering and management; 2) compatibility: the system ensures optimal use by the most popular browsers, such as Internet Explorer and Mozilla Firefox; 3) multi-centric management: the system will be accessible from different centres with diversified access codes; 4) users’ management: the application will enable use from authorized users according to their profile. There will be two types of profile: user profiles and admin profiles. The former profile will be used to perform simple data entries, while the admin profile will have full access to the details of the system and advanced features; 5) advanced functions: control of entered data with ability to view the main summary information and export to CSV/EXCEL format for subsequent statistical studies. These functions enable a constant monitoring of the work evolution by centralizing data and enabling real-time and accurate assessment of the study course, solicitation of the participants, and correction of possible errors.

### Ethical considerations

After a full explanation of the nature of the study, subjects will be asked to participate in the study, and if they accept, they will be asked to sign the informed consent form. Subjects will be ensured that all data remain anonymous. Intelligence about the participation of the patient will not be included in their medical record. Identification codes will be assigned to each subject in order to protect their confidentiality and ensure all data remain anonymous. The subjects may withdraw from the study at any time. The study protocol has been approved by the ethics committee of the coordinating Centre (Bologna) and has been submitted to each local ethics committee (Table 1).

### Commercial and patent rights - dissemination of results

Patients’ rights, and other forms of economic utilization of research results, are considered indisputable rights of the study. It is also considered right of the study to use the data acquired in seminars, conferences, and scientific publications.

### Expected side effects and possible contraindications

There are no predictable side effects that can be attributed to the participation in this study. Patients will continue to receive their usual therapies, and any adverse events that occur during the administration of therapeutic procedures will be notified to the authorities in accordance with the applicable laws.

### Ethical evaluation

The research study described does not present any particular ethical problems.

## Discussion

### Expected results and clinical implications

The study will gather relevant information on the socio-demographic and clinical features, and pathways to care of migrants with first-episode psychosis in Italy. The comparison with native patients will highlight specific risk factors for psychosis in migrants. As a future perspective, it would be an interesting additional research field a case–control study with migrants who, having been recruited in the same catchment areas at the same period of data collection, have not developed psychosis. Finally, the inclusion of mental health centres located in different social and geographic areas will allow inter-regional comparisons of risk factors for psychosis in migrants. Selected centres will be able to obtain more in-depth information on both individual and area-specific social risk factors, as well as an evaluation of the family burden of patients with a psychotic onset. We expect that the results of this study will contribute to the development of prevention programs for psychosis in migrants and will help to improve the effectiveness of early intervention treatments within migrant population.

## Competing interests

The authors declare that they have no competing interests.

## Authors’ contributions

IT is the network main coordinator, the main study-designer and manuscript writer. AF is a network co-coordinator and has significantly collaborated to the study design and the manuscript draft. MB is a network co-coordinator and has collaborated to the data collection and the manuscript draft. VB, BC, LF, MN, MR, OT, PZ and DB are responsible for the coordination of recruitment and assessment in the local centres, they have also advised on the development of the design and have given the final approval of this version to be published. FA, GB, AB, AC, SD, VDV, CDR, SF, MAF, CG, CLC, ML, AM, FP, DP, CR, ET and AV are responsible for the recruitment, the clinical assessment and the manuscript revision. MCD is our Information Technology Supervisor, he has created the online database and will perform statistical analyses. All authors read and approved the final manuscript.

## Pre-publication history

The pre-publication history for this paper can be accessed here:

http://www.biomedcentral.com/1471-244X/14/186/prepub
